# Primary Tumor Size and Tumor–Vessel Interface Following Capecitabine and Temozolomide for Pancreatic Neuroendocrine Tumor

**DOI:** 10.3390/curroncol33020111

**Published:** 2026-02-12

**Authors:** Jin Guo, Kever A. Lewis, Laura Prakash, Priya Bhosale, Ajaykumar Morani, Matthew H. G. Katz, Ching-Wei D. Tzeng, Naruhiko Ikoma, Rebecca Snyder, Michael P. Kim, Chandrikha Chandrasekharan, Arvind Dasari, James C. Yao, Jeffrey E. Lee, Jessica E. Maxwell, Daniel M. Halperin

**Affiliations:** 1 New York Cancer & Blood Specialists, New York, NY 11776, USA; 2 Department of Surgical Oncology, University of Texas MD Anderson Cancer Center, Houston, TX 77030, USAcdtzeng@mdanderson.org (C.-W.D.T.);; 3 Department of Abdominal Imaging, University of Texas MD Anderson Cancer Center, Houston, TX 77030, USA; 4 Department of Gastrointestinal Medical Oncology, University of Texas MD Anderson Cancer Center, Houston, TX 77030, USA; 5 Department of Hematology & Medical Oncology, Winship Cancer Institute of Emory University, Atlanta, GA 30322, USA

**Keywords:** pancreas neuroendocrine tumors, capecitabine, temozolomide, surgical management

## Abstract

Capecitabine/temozolomide (CAPTEM) is an established regimen for patients with metastatic pancreatic neuroendocrine tumors (PanNET) that is being increasingly used for tumor volume reduction in patients with borderline anatomically resectable disease. We sought to understand the response of the primary tumor, defined as changes in the tumor–vascular interface (TVI). This is a retrospective, single-institution study of patients with locally advanced or metastatic PanNET treated with CAPTEM between 2010 and 2020. RECISTv1.1 measurements and TVI assessments of the primary tumor were performed on pre- and post-therapy images. Patients with locally advanced or metastatic PanNET at presentation (*n* = 47) were included. CAPTEM was given for a median of 11 cycles. The most common site of metastatic disease was the liver (*n* = 38). An objective radiographic response in the primary tumor was observed in 6.4% (95% CI 1.7–18.6%) with clinical benefit in 70.2% (95% CI 54.9–82.2%). TVI was modified from >180° to ≤180° in 16.2% (95% CI 6.0–45.5%). Paired analysis of patients pre- and post-CAPTEM did not demonstrate a statistically significant shift in TVI with treatment (*p* = 0.134). A total of four patients had a change from an unresectable primary tumor to an anatomically resectable tumor following CAPTEM. In patients with locally advanced or metastatic PanNET, treatment with CAPTEM is associated with low radiographic response rates and changes in TVI. The degree to which these changes may correlate with surgical resection rates or R0 resections is not known. Extending these investigations in a cohort of PanNET patients offered CAPTEM for neoadjuvant intent could be helpful to understand whether these phenomena persist in that context.

## Simple Summary

Pancreas neuroendocrine tumors can be treated with a combination of chemotherapy agents called capecitabine and temozolomide. While this treatment can be associated with tumor shrinkage, we were interested to know if treatment could also be associated with a response around nearby blood vessels, often a limiting factor in primary tumor resection. Of patients treated with this drug combination, only 16.2% had changes in the vascular relationship of the tumor to the blood vessels. The utility of radiographic response or primary tumor association with the mesenteric blood vessels as a marker of potential resectability is thus not known.

## 1. Introduction

Pancreatic neuroendocrine tumor (PanNET) is a rare gastrointestinal malignancy originating from the neuroendocrine cells of the pancreas with a yearly incidence of approximately 1.25 per 100,000 in the United States [1]. The incidence of PanNET has been steadily rising over time due to earlier detection rates and the increasing use of cross-sectional imaging, yet more than half of patients present with metastatic disease, commonly involving the liver, lymph nodes, or bone [1,2,3,4]. Prognosis for patients with PanNET is strongly associated with tumor stage and histologic grade [1]. However, surgical extirpation is still a central component of curative-intent therapy as resection of the primary PanNET, along with cytoreduction of metastatic sites, has been associated with improved overall survival [1,5,6,7,8,9]. For many patients with PanNET, surgical removal of the primary tumor is constrained by anatomic feasibility, often due to involvement of critical mesenteric vasculature [10].

Minimizing the extent of tumor involvement with vessels such as the main portal vein (PV), superior mesenteric vein (SMV), splenic vein (SV), and superior mesenteric artery (SMA) is a surgically relevant anatomic consideration for patients undergoing pancreatectomy. The concept of tumor–vessel interface (TVI) has been most rigorously studied in patients with pancreatic adenocarcinoma. In this setting, the degree of circumferential mesenteric vascular involvement correlates with rates of microscopically negative resection (R0) and other oncologic outcomes such as recurrence-free and overall survival [11]. While PanNET differs biologically from pancreatic adenocarcinoma, surgical decision-making frameworks in both diseases are governed by shared anatomic constraints. As such, TVI represents a pragmatic, anatomy-based framework for evaluating local tumor extent in patients with PanNET.

Because of this, there has been a growing interest in systemic therapies for patients with PanNET that may reduce local tumor burden and TVI. Capecitabine and temozolomide (CAPTEM) is an established regimen in patients with metastatic PanNET that is associated with meaningful radiologic response rates and durable disease control [12,13,14]. In patients with localized disease, CAPTEM has also been evaluated as a neoadjuvant therapy option, and although these studies show encouraging radiologic response and high rates of surgical resection, conclusions are limited by the retrospective study design and sample size [15,16,17].

Despite these efforts, there remains limited evidence regarding the impact of CAPTEM on TVI. Thus, we sought to evaluate radiographic response and anatomic changes, particularly those related to TVI, in patients with PanNET who were treated with CAPTEM.

## 2. Materials and Methods

### 2.1. Patients

The Institutional Review Board of The University of Texas MD Anderson Cancer Center approved the data collection and analysis for this study. From our institutional multidisciplinary pancreas database (IRB RCR01-112), we identified all patients with PanNET treated with CAPTEM between 2010 and 2020. Patients who had received prior therapy with a somatostatin analog or other systemic chemotherapy were also included. We excluded patients who had their pancreatic primary tumor resected prior to treatment with CAPTEM or had poorly differentiated neuroendocrine carcinomas.

### 2.2. Systemic Therapy

Patients included received capecitabine 750 mg/m^2^ on days 1–14 and temozolomide 200 mg/m^2^ on days 10–14 every 28 days. The duration of treatment was recorded for each patient. Toxicity and dose reduction documentation were obtained from outside medical records where available; however, due to the retrospective nature of the study, adverse events could not be rigorously collected.

### 2.3. Radiologic Evaluation

Computed tomography (CT) imaging was obtained during the pancreatic parenchymal, portal venous, and delayed phases of contrast enhancement using 2.5 mm slice reconstructions. Approximately 125–150 mL of Omnipaque 350 was administered at 4–5 mL/s to achieve an injection duration of approximately 30 s. Bolus tracking was performed with a threshold of a 100-HU increase in the abdominal aorta at the level of the celiac axis. A diagnostic delay of 20 s was applied, resulting in image acquisition approximately 40 s after contrast injection. The scan duration was approximately 5–7 s. The portal venous phase was acquired 20 s after the pancreatic phase using a standardized delay, and the delayed phase was acquired 120 s after injection of contrast. Water was used as a negative oral contrast agent for all phases. All three imaging phases were reviewed using the Philips iSite picture archiving and communication system (Eindhoven, Netherlands) to assess vascular involvement and tumor size.

The pretreatment CT images were reviewed for this study by board-certified abdominal diagnostic imaging faculty (PB and AM) who were blinded to each patient’s clinical history. All patients underwent baseline CT prior to initiation of treatment and were restaged every 8 to 12 weeks. Post-treatment CT was performed within 3 months of completion of CAPTEM therapy.

Treatment response was assessed by comparing the change in primary tumor dimension and by determining the TVI between the primary and mesenteric vessels pre- and post-CAPTEM. Categorization of changes in the primary tumor size was classified using Response Evaluation Criteria in Solid Tumors version 1.1 (RECISTv1.1) system [18]. Progression of disease (PD) was defined as ≥20% increase in the diameter of the primary tumor. Partial response (PR) was categorized as ≥30% decrease in the primary tumor. A complete response (CR) was defined as the disappearance of the primary tumor. Stable disease (SD) is no PR, PD, or CR. The objective response rate (ORR) was defined as the proportion of patients who have a PR or CR following therapy. The clinical benefit rate (CBR) was the proportion of patients who achieved a CR, PR or SD following therapy.

The TVI describes the circumferential relationship between the primary tumor and mesenteric vasculature. Measured on axial images, the TVI was determined for the primary tumor and the PV, SMV, SV, SMA, common hepatic artery (CHA), and splenic artery (SA). In each case, the TVI was determined pre- and post-CAPTEM, and categorized as no involvement (absence of tumor contact, with intervening normal pancreas or fat), ≤180° abutment, or ≥180° encasement. TVI was evaluated at both the vessel level and the patient level. At the patient level, overall vascular involvement was summarized using the worst TVI category observed across all assessable vessels at both pre- and post-CAPTEM timepoints. Change in TVI was defined by comparing pre- and post-CAPTEM imaging: improvement was defined as a decrease in the worst TVI category, progression as an increase, and no change as an unchanged category. Vessels that were occluded or thrombosed on imaging were considered not assessable for TVI measurement and were excluded from paired analyses of TVI change.

### 2.4. Evaluation of Anatomic Feasibility of Resection

Post hoc surgical review of all cases was performed by a board-certified surgical oncologist with extensive experience in resection of PanNET (JEM) using CT imaging pre-CAPTEM and post-CAPTEM. The standardized definitions of anatomic resectability at MD Anderson Cancer Center were used to classify the anatomic stage of each primary tumor into anatomically resectable, borderline resectable, or locally advanced at each timepoint [19]. Each case was further classified based on the feasibility of resecting both the primary tumor and at least 70% of the metastatic tumor burden at other sites. These cases were classified as overall resectable, overall borderline resectable or overall unresectable, and this was done to account for the radiographic effect CAPTEM may have had on the patient’s metastatic disease burden in addition to that of the primary tumor.

### 2.5. Clinicopathologic and Histologic Data

Clinicopathologic data retrieved from the institutional database included patient sex, age, stage, Ki-67 proliferation index, World Health Organization (WHO) tumor grade, serum tumor markers, and metastatic sites. Clinical stage was based on the 8th edition of the American Joint Committee on Cancer staging manual [20].

### 2.6. Statistics

Summary statistics were used to describe patient and tumor characteristics. Categorical variables are reported as counts and proportions, and continuous variables as medians with ranges. Exact 95% confidence intervals for proportions were calculated using binomial distributions.

TVI was analyzed as a paired categorical variable. At the patient level, TVI was summarized using the worst TVI category across assessable vessels at each timepoint. Changes in high-risk vascular anatomy, defined as ≥180° encasement, were evaluated using paired comparisons. McNemar’s test was used to assess whether CAPTEM was associated with a systematic shift in patients with TVI ≥ 180° from baseline to post-treatment imaging. Effect sizes are reported as absolute proportions with 95% confidence intervals. Standard Kaplan–Meier methods were used for survival estimates. Overall survival (OS) was defined as the interval from the start of CAPTEM therapy until the date of death or last follow-up. Progression-free survival (PFS) was defined as the interval from the start of CAPTEM therapy until documented radiologic disease progression with censoring at the date of death or last follow-up.

## 3. Results

### 3.1. Patient Demographics

We identified 47 patients with PanNET who received CAPTEM at The University of Texas MD Anderson Cancer Center between 2010 and 2020. Clinicopathologic features are summarized in Table 1. A total of 42 (89%) patients had metastatic disease at presentation. Of those, 39 (93%) had liver involvement, 2 (5%) lung, and 2 (5%) peritoneal. Multiple organ sites were involved in 21% (9/42) of patients. Most patients had World Health Organization (WHO) grade 2 tumors (57%), with the remainder having grade 1 (34%) or grade 3 (8%) tumors. The primary tumor was located within the head of the pancreas in 11 patients (23%) and body/tail in 36 (77%) patients.

### 3.2. Treatment and Safety

CAPTEM was given in the frontline setting for 27 (57%) patients. The remaining patients had received prior treatment with somatostatin analogs (*n* = 14), everolimus (*n* = 8), surufatinib (*n* = 4), and/or combination 5-fluorouracil, doxorubicin, and streptozocin (FAS; *n* = 6). Patients that received CAPTEM were given a median of 11 (range, 2–34) cycles of CAPTEM prior to radiographic evaluation of response. The majority of patients received CAPTEM locally under the care of a non-MD Anderson medical oncologist, and thus adverse event reporting was limited. Overall, CAPTEM was generally well tolerated, and no unexpected safety signals were observed given the data limitation. Dose reduction occurred in 3 out of 36 patients with available dosing data. Two of the patients required dose reduction due to thrombocytopenia, while the other patient required dose reduction for renal dysfunction. Grade 3 nausea and vomiting occurred in one patient, which prompted discontinuation of treatment.

### 3.3. Efficacy

**Figure 1:**
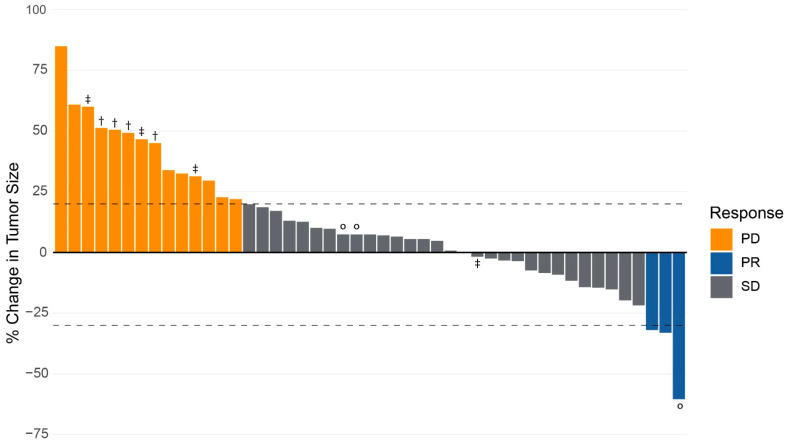
Radiographic response of primary tumor in response to capecitabine and temozolomide in patients with neuroendocrine tumor of the pancreas. Response measured as the percentage change in the diameter of primary tumor from baseline measurement using RECISTv1.1 criteria. Progression of disease (PD) is defined as a ≥20% increase in the diameter of the primary tumor. Partial response (PR) is categorized as ≥30% decrease in the primary tumor. A complete response (CR) is defined as the disappearance of the primary tumor. Stable disease (SD) is no PR, PD, or CR. Key: ° = patients undergoing surgical resection. † = patients with improvement in tumor vessel interface of primary tumor following CAPTEM therapy. ‡ = patients with improvement in anatomic resectability of primary tumor following CAPTEM therapy.

After treatment with CAPTEM, 30 (64%) patients achieved SD in the primary tumor by RECISTv1.1 criteria. PR was achieved in 3 (7%) patients, and 14 (30%) had PD while on CAPTEM therapy. No patients had a CR. The ORR was 6.4% (95% CI 1.7–18.6%), and the CBR was 70.2% (95% CI 54.9–82.2%). A waterfall plot of the radiographic response of the primary tumor to CAPTEM chemotherapy is provided in Figure 1.

### 3.4. Tumor Vessel Interface

A total of 31 patients had measurable TVI prior to CAPTEM treatment, with 19 (61%) having ≥180° involvement and the remaining 12 (39%) having <180° involvement. A total of 28 (90%) patients had venous involvement, most commonly the splenic vein (*n* = 20). Arterial involvement was observed in 19 (61%) patients pre-CAPTEM treatment, with involvement of the CHA observed in 12 patients, SA in 10 patients, and SMA in 8 patients.

Of the 19 patients with at least one vessel demonstrating ≥180° involvement prior to CAPTEM treatment, 4 patients improved to a TVI of <180° after treatment (response rate 21.0%, 95% CI 6.0–45.5%). Paired analysis of patients pre- and post-CAPTEM did not demonstrate a statistically significant shift in TVI with treatment (*p* = 0.134). No patients had worsened TVI after CAPTEM treatment.

Of the four patients that had improved TVI with CAPTEM, all 4 had primary tumors within the body/tail of the pancreas (Table 2). All four patients had improvement of >180° encasement of the splenic artery to ≤180° involvement. Only 2 had pre-CAPTEM splenic vein involvement that improved with therapy. A single patient had superior mesenteric vein and hepatic artery involvement that improved with CAPTEM. None of these patients were referred to a surgeon for consideration of an operation because their metastatic burden, tumor biology, fitness, or some combination of those factors were considered by the medical oncologist to be prohibitive.

### 3.5. Anatomic Feasibility of Primary Tumor Resection

A total of 44 (94%) patients had imaging sufficient for evaluation of primary tumor anatomy both pre- and post-CAPTEM therapy. Prior to CAPTEM, 23% (*n* = 11) of patients had anatomically unresectable primary tumors, 21% (*n* = 10) had borderline resectable tumors, and 51% (*n* = 24) had anatomically resectable primary tumors. After CAPTEM therapy, 4 patients (9%) had improvement in the anatomic resectability of the primary tumor. Three patients were downstaged from unresectable to borderline resectable (*n* = 1) or anatomically resectable (*n* = 2). The final patient had a conversion of a borderline resectable primary tumor to an anatomically resectable tumor due to tumor size. None of these patients had changes in TVI with CAPTEM therapy.

Of the 47 patients included in this study, 3 (6%) underwent surgical resection (Table 3). All three patients achieved stable disease in the primary tumor as their best RECISTv1.1 response and demonstrated no change in TVI following CAPTEM. Two patients underwent pancreaticoduodenectomy with R0 resection, and one underwent combined distal pancreatectomy, splenectomy, and left partial hepatectomy. In the patients that underwent surgical resection, a median of 11 cycles of CAPTEM was received (range 5–22).

### 3.6. Survival

Median follow-up for the cohort was 44.7 months. The mortality rate was 48.9% with a median overall survival of 41.3 months (95% CI 36.7–75.8 months). After initiation of CAPTEM therapy, 63.8% had disease progression with a median progression-free survival of 19.9 months (95% CI 11.5–39.6 months).

## 4. Discussion

In this analysis of patients with locally advanced or metastatic PanNET treated at a tertiary referral center, we sought to evaluate the impact of CAPTEM on the radiographic response of the primary tumor using RECISTv1.1 and the anatomic feasibility of primary tumor resection using TVI and subjective assessment. CAPTEM was associated with a low radiographic response rate at 6.4% and a low rate of improved TVI at 21.0% in those with >180° TVI prior to treatment. When evaluating the anatomic feasibility of primary tumor resection, only 9% of patients had improvement following CAPTEM treatment. Thus, rates of radiographic treatment response to CAPTEM therapy in patients with locally advanced or metastatic PanNET may not be associated with meaningful improvements in TVI or anatomic feasibility of primary tumor resection.

Perioperative chemotherapy is increasingly studied in patients with PanNET with the intent of improving the anatomic feasibility of resection, reducing the rate of postoperative recurrence, and potentially improving survival [5,6,8,17,21,22,23,24,25]. In a study of six patients, CAPTEM given with neoadjuvant intent with or without radiotherapy resulted in two partial responses and four cases of disease stabilization [16]. Squires et al. evaluated patients with locally advanced or borderline resectable metastatic PanNET with or without intact primary tumor and demonstrated radiologic response rates of 43% and concluded that CAPTEM may improve rates of surgical resection [17]. In ECOG ACRIN E2211, treatment with CAPTEM in patients with advanced PanNET achieved an overall disease response rate of 40% [15]. In our study, the rate of primary tumor response was only 6.4%; however, clinical benefit remained high at 70.2%.

Systemic therapy in PanNET has historically been reserved for patients with metastatic disease or progressing, unresectable locally advanced tumors. In contemporary practice, however, systemic therapy is increasingly employed in patients with locally advanced or borderline resectable disease with the goal of improving the likelihood of an oncologically meaningful operation. This is an approach adapted from pancreatic adenocarcinoma and implicitly adopts not only the use of systemic therapy but also the broader surgical-decision-making framework in which resectability uses a tripartite assessment of anatomic feasibility, disease biology, and patient fitness for surgery [26]. This systematic evaluation of a patient’s disease state may allow for more nuanced surgical decision-making, but the adaptation of this framework from pancreas adenocarcinoma to PanNET patients in terms of its impact on resection rates, surgical outcome, and survival has not been rigorously evaluated.

Despite its limitations, within this framework, anatomic feasibility is driven less by tumor size and more by the relationship between the primary tumor and surrounding mesenteric vasculature, suggesting that TVI may be a clinically meaningful radiographic surrogate for technical resectability of the primary tumor in patients with PanNET. Thus, therapy associated with ORR, and by extension improvement in TVI, may be beneficial in patients who could be considered for surgical resection or cytoreduction.

In our cohort, CAPTEM was not associated with meaningful improvement in TVI or rates of anatomically feasible resection of the primary tumor. Although a small number of patients demonstrated conversion from radiographically unresectable to technically resectable tumors or had an improvement in TVI, most patients were never referred for surgical evaluation due to extensive metastatic disease. The use of a metastatic or locally advanced cohort therefore allowed for assessment of both radiographic response and effect on mesenteric vasculature to CAPTEM on the primary pancreatic tumor, independent of surgical selection, intent, or timing.

The findings of this study also raise a broader question regarding the role of TVI as a surrogate marker of response and resectability in PanNET. In pancreatic adenocarcinoma, changes in TVI are often interpreted as evidence of treatment-induced biologic response and are linked to anatomic feasibility of pancreatic resection. Whether this relationship holds true in PanNET is less certain. Specifically, it is unclear whether a lack of improvement in TVI with CAPTEM means that CAPTEM is less efficacious at improving TVI or if TVI is not a reliable marker for the true treatment effect of CAPTEM. While this study is underpowered to make robust conclusions about treatment efficacy with CAPTEM, extrapolating TVI-based response paradigms from pancreatic adenocarcinoma to PanNET should be approached cautiously until prospective, disease-specific data clarify the true radiographic response with CAPTEM given with neoadjuvant intent.

This study has several limitations. Adverse event data were incompletely captured in this retrospective and single-center cohort, limiting the ability to assess treatment-related toxicity or to contextualize radiographic findings within a risk–benefit framework. The metastatic and locally advanced nature of the cohort also precludes conclusions regarding overall resectability and surgical decision-making, given the inability to reliably assess patient fitness and case mix of our cohort. Finally, the modest sample size limits the power to detect small but clinically meaningful anatomic changes and restricts subgroup analyses.

Overall, in this cohort of patients with metastatic and locally advanced PanNET, meaningful changes in the radiographic response of the primary tumor after CAPTEM did not correlate with changes in vascular involvement or anatomic feasibility of pancreatic resection. Utilizing change in TVI as a surrogate for reliable treatment response within the primary tumor after CAPTEM should be used cautiously, and future studies should work to understand the potential for and effect on surgical resectability of changes in primary tumor response after CAPTEM therapy given with neoadjuvant intent.

## Figures and Tables

**Table 1:** Baseline patient characteristics.

Age at time of biopsy, median (range)		62 (24–77)
Gender		
	Female	17 (36)
	Male	30 (64)
Race/Ethnicity		
	White	43 (92)
	Asian	1 (2)
	Black	1 (2)
	Hispanic or Latino	1 (2)
	Other	1 (2)
Stage at diagnosis		
	Localized	5 (11)
	Metastatic	42 (89)
WHO Grade		
	Grade 1	16 (34)
	Grade 2	27 (57)
	Grade 3	4 (8)
Treatment prior to CAPTEM		
	Treatment naïve	27 (57)
	Prior SSA	14 (30
	Other ^a^	6 (13)
Primary Tumor Location		
	Head	11 (23)
	Body/Tail	36 (77)

WHO = World Health Organization; CAPTEM = Capecitabine and temozolomide; SSA = Somatostatin Analogues. ^a^ 3 patients received surufatinib, 2 everolimus, and 1 ziv-aflibercept.

**Table 2:** Patients with improvement in tumor vessel interface after treatment with CAPTEM.

Tumor Location	Stage at Presentation	Duration of CAPTEM (Cycles)	RECISTv1.1 Response	Tumor Vessel Interface		Anatomic Resectability of Primary Tumor		Contraindications to Surgical Resection
				Pre	Post	Pre	Post	
Body/Tail	Metastatic	11	PR	SMV, PV abutment; CHA, SV, SA encasement	PV, CHA, SV, SA abutment	Unresectable	Unresectable	Extent of vascular involvement
Body/Tail	Metastatic	21	PR	SA encasement	SA abutment	Anatomically Resectable	Anatomically Resectable	Extent of hepatic metastases
Body/Tail	Metastatic	11	PR	SV, SA encasement	SV, SA encasement	Anatomically Resectable	Anatomically Resectable	Extent of hepatic metastases
Body/Tail	Metastatic	32	PR	SA encasement	SA abutment	Anatomically Resectable	Anatomically Resectable	Extent of hepatic metastases

CAPTEM = capecitabine and temozolomide; PR = partial response; SMV = superior mesenteric vein; SV = splenic vein; PV = portal vein; SA = splenic artery; CHA = common hepatic artery.

**Table 3:** Patients who underwent surgical resection after treatment with CAPTEM.

Tumor Location	Stage at Presentation	Duration of CAPTEM (Cycles)	RECISTv1.1 Response	Time to Resection (Months)	Tumor Vessel Interface		Anatomic Resectability of Primary Tumor		Operation	Primary Tumor Resection Margin
					Pre	Post	Pre	Post		
Body/Tail	Metastatic	14	SD	3	SV Occlusion		Anatomically Resectable		Distal pancreatectomy with splenectomy; Left hepatectomy with MWAx3 to right liver lesions	R0
Head	Metastatic	22	SD	1	No involvement		Anatomically Resectable		Pancreaticoduodenectomy with planned liver transplant given a near complete response in liver	R0
Head	Locally Advanced	5	SD	2	SMV, PV, and SMA abutment		Borderline Resectable		Pancreaticoduodenectomy with SMV resection (primary repair)	R1

CAPTEM = capecitabine and temozolomide; SD = stable disease; SMV = superior mesenteric vein; PV = portal vein. SMA = superior mesenteric artery; MWA = microwave ablation.

## Data Availability

The data presented in this study are available on request from the corresponding authors.

## Abbreviations

The following abbreviations are used in this manuscript:

CAPTEM	Capecitabine/temozolomide
PanNET	Pancreas neuroendocrine tumor
TVI	Tumor–vessel interface
PV	Portal vein
SMV	Superior mesenteric vein
SV	Splenic vein
SMA	Superior mesenteric artery
PFS	Progression free survival
OS	Overall survival
ORR	Objective response rate
PD	Progression of disease
SLD	Sum of the longest diameters
PR	Partial response
CR	Complete response
SD	Stable disease
CBR	Clinical benefit rate
WHO	World Health Organization
